# Chiral metamaterials: enhancement and control of optical activity and circular dichroism

**DOI:** 10.1186/s40580-015-0058-2

**Published:** 2015-11-09

**Authors:** Sang Soon Oh, Ortwin Hess

**Affiliations:** The Blackett Laboratory, Department of Physics, Imperial College London, Prince Consort Road, London, SW7 2AZ UK

**Keywords:** Chirality, Metamaterial, Optical activity, Circular dichroism, Ellipticity

## Abstract

The control of the optical activity and ellipticity of a medium has drawn considerable attention due to the recent developments in metamaterial design techniques and a deeper understanding of the light matter interaction in composite metallic structures. Indeed, recently proposed designs of metaatoms have enabled the realisation of materials with unprecedented chiral optical properties e.g. strong optical activity, broadband optical activity, and nondispersive zero ellipticity. Combining chiral metamaterials with nonlinear materials has opened up new possibilities in the field of nonlinear chirality as well as provided the foundation for switchable chiral devices. Furthermore, chirality together with hyperbolicity can be used to realise new exciting materials such as photonic topological insulators. In this review, we will outline the fundamental principles of chiral metamaterials and report on recent progress in providing the foundations for promising applications of switchable chiral metamaterials.

## Introduction

Controlling the polarisation states of light forms an important part of classical optics. Remarkably, the journey to understand and exploit polarisation states of light waves started in the very early days of classical optics, as exemplified by Malus’s law, and the discovery of birefringent effects in calcite and sodium chloride crystals. Even today, in modern photonics, it is just as important to control both linear and circular polarisation states, particularly where an exact and subtle control of light waves has led to a variety of applications and new areas of research. Unsurprisingly, considerable progress has been made, particularly as many exciting new ideas are being proposed in association with recently emergent fields of metamaterials and nanoplasmonics.

Optical activity, the rotation of polarisation of a linearly polarised light beam, was first discovered by F. J. Arago in 1811 in sunlight that had transmitted a quartz crystal placed between crossed polarisers [1, 2]. Since then, it has been exploited in many disciplines of science including chemistry, physics, and biology. Despite of the long development period of chiral material research, there is still high demand for rotatory power control in modern fields such as optoelectronics, life science microscopy, displays, and photography [3].

In media with a chiral structure (chiral media), optical activity is a result of the coupling between electric and magnetic fields [4]. Natural chiral materials such as quartz, amino acids, and sugars, have weak electro-magnetic coupling and optical activity is thus a rather weak effect compared to dielectric polarisation effect. In general, optical activity and dielectric polarisation effects are characterised by the chirality parameter *κ* and the refractive index *n*. However, it was shown that a “chiral metamaterial” could bring about a dramatic increase in the chirality parameter and make it even comparable in magnitude to the refractive index, thereby leading to a negative refractive index without requiring any negative permittivity or negative permeability [5]. A metamaterial is an artificial composite structure engineered to have properties not present in nature and it is called a chiral metamaterial when it is designed to have unnatural chiral parameters. The increase of the chirality parameter is possible since chiral metamaterials can dramatically enhance the electro-magnetic coupling through specially arranged chiral metallic structures rather than using naturally occurring chiral materials [3]. Accordingly, significant efforts have been devoted to find a material with strong optical activity over the past few years [6–10]. The proposed chiral metamaterials were shown to have optical activities at least two orders of magnitude greater than natural materials [11] and some even show a negative refractive index [12].

On the other hand, one of the advantages of metamaterials is the tunability of their optical properties through geometric and material parameters. For example, the refractive index of a metamaterial can be made negative [13] or extremely large [14] by controlling electric/magnetic resonances or couplings. Furthermore, it is possible to have dynamic control over the optical properties, for example by optically tuning the resonance frequencies of a split ring resonator (SRR) array on a semiconductor substrate [15], or gate controlled transmission in graphene metamaterials [16]. Furthermore, by controlling resonances in chiral metamaterials with optical pumping, the sign of the circular dichroism can be reversed, which is impossible in naturally occurring chiral materials [17]. Adding to recent review papers on chiral metamaterials, discussing, for example, simulations and experiments of chiral metamaterials [18, 19], and chirality of plasmonic nanostructures [20] we will summarise recent achievements in chiral metamaterial research and particularly focus on new functionalities such as switching and control of light polarisations.

## Review

In this section, we will layout the underlying physics of chiral media and review recent advances in the research of chiral metamaterials. After discussing chirality-related optical parameters based on wave propagation in chiral media and the effective medium approach we will examine how optical activity can be enhanced through chiral metamaterials. Subsequently, we will explain how circular dichroism can be controlled in chiral media and finally we will review recent papers on switchable chirality.

### Theory of optical chirality

An object is called chiral if there is no translational or rotational transformation that allows its mirror image to be superimposed onto the original one. As Lord Kelvin mentioned [21], chiral media and systems are ubiquitous. For example, our hands, screws, shells of snails and so on are clearly chiral. Additionally, at molecular scales, there are a variety of chiral molecules like amino acids and sugars. Very frequently, however, a medium’s chirality is ignored, which has led, for example, to the disastrous misuse of sedative drug thalidomide [22]. From the perspective of optics, there are two main reasons for this: the optical response due to chirality is in general weaker than the achiral response and a medium can include an equal number of elements with two different handedness (which is called racemic mixture) rendering it optically inactive.

**Fig. 1:**
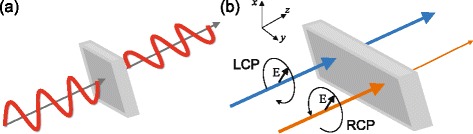
Optical activity and circular dichroism in chiral medium. **a** Optical activity: the electric field vector of linearly polarised light rotates around the axis parallel to its propagation direction (+*z*) while passing a chiral medium. **b** Circular dichroism: transmissions for RCP and LCP waves are different to each other due to difference in absorptions between RCP and LCP waves. At a fixed point in space, the electric field vector of RCP (LCP) wave rotates around the *z* axis in clockwise (anticlockwise) direction when the observer is facing into the oncoming wave

Optical activity and circular dichroism are characteristic (but different) manifestations of the optical response of chiral media. As mentioned previously, optical activity relates to the rotation of the plane of polarisation of a linearly polarised light beam, while circular dichroism denotes the difference in absorption of light with left or right circular polarisation (see Fig. 1). The optical activity is described by the rotation angle degree *θ*. As we will see in the following section, it can be related to the real part of the chirality parameter, which is an intrinsic material property. In the same manner, circular dichroism, which is measured by the difference in absorption, is related to the imaginary part of the chirality parameter. Since circular dichroism depends on transmission, it is convenient to use the ellipticity, which is a function of the relative difference in transmission. In the following, we will outline the derivation of expressions that relate optical activity *θ* and ellipticity *η* to the real and imaginary parts of the chirality parameter *κ*, starting from the constitutive relation of a chiral medium. Here we should note that optical rotatory dispersion, the unequal rotation of the plane of polarisation of light of different wavelengths, is different from optical activity.

#### Chirality parameter and ellipticity

Chiral media are a subset of bi-isotropic media where the electric and magnetic fields of light waves are coupled to each other. Thus, the electromagnetic or optical response of bi-isotropic media can be described by the following relations:

(1) $$\begin{array}{@{}rcl@{}} \left(\begin{array}{c} \mathbf{D} \\ \mathbf{B} \end{array} \right) = \left(\begin{array}{c c} \varepsilon_{0} \varepsilon & (\xi + i \kappa) \sqrt{\mu_{0} \varepsilon_{0}} \\ (\xi - i \kappa) \sqrt{\mu_{0} \varepsilon_{0}} & \mu_{0} \mu \end{array}\right) \left(\begin{array}{c} \mathbf{E} \\ \mathbf{H} \end{array}\right) ~,  \end{array} $$

where *ε* is the relative permittivity of the medium, *ε*_0_ is the permittivity of vacuum, *μ* is the relative permeability of the medium, and *μ*_0_ is the permeability of vacuum. The Tellegen parameter *ξ* is a dimensionless magneto-electric parameter which describes the reciprocity of the material. When the material is nonreciprocal (reciprocal), *ξ*≠0 (*ξ*=0). For chiral materials, the chirality parameter is *κ*≠0. In this paper we will consider reciprocal (*ξ*=0) and chiral (*κ*≠0) media, also known as Pasteur media [18].

Circular polarisation states are eigenstates of chiral media. This can be shown using Maxwell’s equation with Eq. (1).

(2) $$\begin{array}{@{}rcl@{}} \nabla \times \mathbf{E} &=& - \frac{\partial \mathbf{B}}{\partial t}, \end{array} $$

(3) $$\begin{array}{@{}rcl@{}} \nabla \times \mathbf{H} &=& \frac{\partial \mathbf{D}}{\partial t}. \end{array} $$

By assuming that all fields of waves in the chiral medium have a plane wave character *e* ^*ikz*−*iωt*^ we can derive the eigenvalue equation which has two solutions for the wavevector *k*:

(4) $$\begin{array}{@{}rcl@{}} \left(\begin{array}{c} k_{+} \\ k_{-} \end{array} \right) = \frac{\omega}{c} \left(\begin{array}{c} \sqrt{\varepsilon \mu} + \kappa \\ \sqrt{\varepsilon \mu} -\kappa \end{array} \right),  \end{array} $$

where *k*_±_ are the wavevectors of right-hand circularly polarised (RCP) and left-hand circularly polarised (LCP) waves. Throughout this paper, we assume that the electric field vector at a fixed point rotates anticlockwise in the RCP wave when looking along the energy propagation direction ($\hat {z}$) as shown in Fig. 1(b). Then, by omitting the common factor *e* ^*ikz*−*iωt*^, RCP wave can be expressed as $\frac {F}{\sqrt {2}} (\hat {x}-i\hat {y})$ and the LCP wave $\frac {F}{\sqrt {2}} (\hat {x}+i\hat {y})$ where *F* is the field amplitude.

The two different wavevector solutions (Eq. (4)) indicate that the two eigenstates propagate with different speeds. Therefore, the effective refractive indices for RCP and LCP waves are different to each other with *n*_+_=*n*+*κ* and *n*_−_=*n*−*κ* where the racemic refractive index *n* is $\sqrt {\varepsilon \mu }$. This difference of refractive indices for circular polarisations is called circular birefringence and leads to rotation of linear polarisation and circular dichroism. Using the refractive indices for RCP and LCP waves, the chirality parameter can be expressed as *κ*=(*n*_+_−*n*_−_)/2. Clearly, the circular birefringence vanishes when *κ*=0. As we will see in the next section, the real part of *κ* describes the rotation of the polarisation ellipse whereas the imaginary part describes the circular dichroism [23, 24].

Additionally, planar chiral metamaterials (PCMs) with single or multiple layers will be considered in the latter parts. Since PCMs are not bi-isotropic chiral media, one should be careful when describing their chiral responses. For example, circular polarisation states are no longer eigenstates of PCMs even though they exhibit optical activity and circular dichroism. Theoretical anlaysis on PCMs can be found in Ref. [25].

#### Homogenisation and effective optical parameters

A key concept of metamaterials is the homogenisation principle, implying that effective optical parameters can be defined for artificially designed composite medium with a suitable subwavelength metaatom size (*a*≪*λ*). This allows one to realise completely new optical properties such as low plasma frequencies [26] and artificial magnetism using split ring resonators [27] which enable us to achieve negative refractive indices [28]. To describe the effective permittivity and permeability for metamaterials slabs, *ε*_*eff*_, *μ*_*eff*_, the effective medium theory from solid state physics has been applied to metamaterials [29, 30]. For the case of chiral metamaterials, the effective chirality parameter *κ*_*eff*_ can be defined in a similar manner as well as the effective refractive indices for different circular polarisation, *n*_+_ and *n*_−_ [18, 31, 32]. Kwon *et al.* derived the relations between the chiral parameters for bi-isotropic metamaterials, and tested the parameter retrieval for a doubly periodic optical chiral metamaterial structure [31]. Homogenisation for multilayer PCMs was examined in connection with coupling between metaatoms [33].

**Fig. 2:**
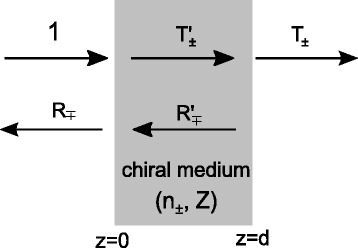
Effective parameters for a chiral medium slab. A chiral medium slab with the thickness *d*. *T*_±_ (*R*_±_) are the transmission (reflection) coefficients for RCP (+) and LCP (-) waves, respectively. For homogeneous chiral media and four-fold symmetric chiral metamaterials, the impedance *Z* is the same for RCP and LCP waves leading to an equal reflection *R*_∓_. Adapted from Ref. [32]

In general, the procedure of calculating effective parameters from transmission and reflection coefficients for a slab is called ‘parameter retrieval’ (see Fig. 2) and the effective chirality parameters *κ*_*eff*_ is given as [32]:

(5) $$\begin{array}{@{}rcl@{}} \operatorname{Re}(\kappa_{eff}) &=& \frac{\arg(T_{+}) - \arg(T_{-}) + 2 m \pi }{2k_{0} d}, \end{array} $$

(6) $$\begin{array}{@{}rcl@{}} \operatorname{Im}(\kappa_{eff}) &=& \frac{\ln(|T_{+}|) - \ln(|T_{-}|)}{2k_{0} d},  \end{array} $$

where *k*_0_ is the wavevector in free space, *d* is the thickness of the metamaterial slab and *m* is an integer determined so that the numerator is in the interval [−*π*,*π*]. *T*_±_ are complex transmission coefficients of RCP and LCP waves, which are defined by $T_{\pm }=E_{\pm }(z=d)/E^{inc}_{\pm }(z=0)$ respectively, where *E*_±_ are the electric field components for RCP (+) and LCP (−) waves and $E^{inc}_{\pm }$ are the electric field components of the incident beam with RCP and LCP.

Since the optical rotation angle *θ* is the phase difference between RCP and LCP waves, we have the following relations.

(7) $$\begin{array}{@{}rcl@{}} \theta = k_{0} d \operatorname{Re}(\kappa_{eff}) \end{array} $$

In addition, the ellipticity is defined by the angle 2*η* whose tangent is equal to the ratio of the minor to the major amplitudes of polarisation ellipse [23, 34], and therefore

(8) $$\begin{array}{@{}rcl@{}} \eta = \frac{1}{2} \arctan \left(\frac{|T_{+}|^{2} - |T_{-}|^{2}}{|T_{+}|^{2} + |T_{-}|^{2}} \right).  \end{array} $$

Using the relation $|T_{+}|/|T_{-}| = e^{2 k_{0} d \operatorname {Im}(\kappa _{eff})}\phantom {\dot {i}\!}$ obtained from Eq. (6), Eq. (8) can be expressed as

(9) $$\begin{array}{@{}rcl@{}} \tan(2 \eta) = \tanh (2 k_{0} d \operatorname{Im}(\kappa_{eff})). \end{array} $$

For *η*≪1, this can be approximated as

(10) $$\begin{array}{@{}rcl@{}} \eta \approx k_{0} d \operatorname{Im}(\kappa_{eff}). \end{array} $$

Therefore, the optical rotation angle and ellipticity are linearly proportional to the real and imaginary part of chirality parameters respectively when the ellipticity is small. Note that this can be justified only when there is no LCP to RCP or RCP to LCP conversion in transmission since we take account of LCP to LCP or RCP to RCP transmission. More insightful interpretation of these relations can be found in Ref. [23].

#### Symmetry consideration: *C*_4_ symmetry, time-reversal and reciprocity

Although a chiral metamaterial does by definition not have any mirror symmetry, it often retains other spatial symmetries such as four-fold rotational symmetry (*C*_4_) [19]. The chirality parameter of a chiral metamaterial can be anisotropic even when its unit cell has an anisotropic geometry, e.g. metallic elements with different length for different directions. In this case, the chirality parameter should be expressed in a tensorial form and there can be a coupling effect between LCP and RCP waves. However, for four-fold symmetric geometries, expressions of the chirality parameter become simplified and the coupling effect, which leads to polarisation conversion, vanishes [31, 32, 35]. On the basis of group theory, Bai *et al.* showed that there is no polarisation conversion in reflection for a single layer gammadion array on a dielectric substrate and no polarisation conversion in transmission for a symmetrically layered chiral metamaterials [25].

In addition, temporal symmetries like time-reversal have been an important topic associated with reciprocity [6]. In 2003, Schwanecke *et al.* reported experimental evidence of broken time-reversal symmetry interaction of light with an artificial nonmagnetic material [36]. Later, however, Kuwata-Gonokami *et al.* showed that optical activity in planar structures does not violate time-reversal symmetry and the polarisation effect is reciprocal [6]. Bai *et al.* demonstrated that the optical activity in PCMs is, indeed, a reciprocal effect.

#### Optical response of chiral metamaterials

Generally, the optical response of a metamaterial is determined by its geometrical properties: size, connectivity and shape of its metallic elements. In fact, for chiral metamaterials, we can list important factors that determine chirality parameters such as: (i) the helicity of metallic elements (straight or helical), (ii) the relative orientation of the metallic elements, (iii) the connectivity over the cell boundary (wire or cut-wire), and (iv) the connectivity between metallic elements inside a unit cell. From these criteria, we can estimate the optical response of chiral metamaterials, the refractive index and chirality parameters. Firstly, the helicity of the constituent metallic elements in a unit cell is directly associated with the strength of chirality of the medium since it determines how the magnetisation is induced by the electric currents flowing through the metallic elements (wires, plates) and how electric polarisation is generated by electric charges in the metallic elements. For example, the chirality parameter of a helical medium has a strong dependence on the radius of helices, radius of the wires and the pitch of the helices [37]. Some insightful guidelines can be found in [20]. Secondly, the relative orientation of the metallic elements strongly affects the resulting Coulomb interactions and electric polarisation allowing changes to the optical activity and circular dichroism. Theoretical model for chiral metallic nanoparticle assemblies can be found in [38]. Thirdly, the connectivity of wires determine the types of electrical resonances, whether its spectra is Lorentzian or Drude. For example, the connected wire mesh array shows Drude type electrical behaviour since the electrons in the wire can move freely in response to the external electric fields, while in disconnected wires, called cut-wire arrays, the electrons are stopped at the end of metallic wires resulting in Lorentzian resonances. Therefore, chiral metamaterials with helical wires [39] show a Drude type spectral response and they can be analysed using the tri-helical model [37]. Lastly, the connectivity inside a unit cell makes the chirality weaker by making current routes of different chirality. The gyroid is a good example of weakened chirality resulting from the connection between metallic elements (see Section 2.4.2 for details) [40]. For an achiral connected wire array metamaterial, the effect of connectivity has been investigated [41], explaining a drastic change in optical parameters of metamaterials.

#### Resonance in chiral metamaterials

**Fig. 3:**
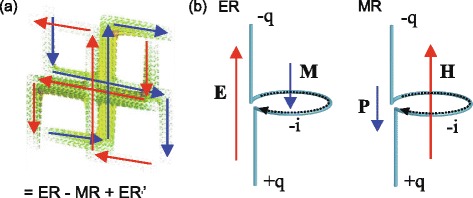
*Ω*-particle model. **a** Current distribution for the lowest frequency resonance at 5.6 GHz in conjugated double-layered gammadion chiral metamaterials. The red (blue) arrows denote the direction of current in the metallic elements in the front (back) layer. The resonance can be understood as a superposition of electrical and magnetic resonances, ER, MR, and ER’. Adapted from Ref. [42]. **b** Schematic figure of *Ω*-particle. (left) Magnetisation **M** is strongly induced by electric field **E** at electrical resonance (ER). (right) Polarisation **P** is strongly induced by magnetic fields **H** at magnetic resonance (MR). *q* and *i* are induced electric charge and current respectively

An essential element in achieving enhanced chiral response (optical activity and ellipticity) is the strong resonance of the constituent metaatoms upon excitation by external electromagnetic waves, resulting in enhanced electric current (electric charge) and leading to strong magnetisation (polarisation). Therefore, we can regard a chiral metamaterial as a structure that exploits electrical or magnetic resonances to enhance or control optical activity and ellipticity. For example, in double layered PCMs, resonances can be classified as electric resonance (ER) and magnetic resonance (MR) [42]. As mentioned in Ref. [42], however, the resonances should be considered as mixed resonances of ERs and MRs for most chiral metamaterials (see Fig. 3(a)).

In the case of a single layer PCMs without a substrate, there is no way to induce magnetisation parallel to the electric field for a normal incidence since the current on one single plane only produces magnetisation along the propagation direction locally and the sum vanishes due to geometrical symmetry. The single layer PCM without a substrate, therefore, does not show optical activity for normal incident waves. However, in the case of a single layer PCM with a substrate on one side, it can generate rotation of polarisation direction leading to optical rotation [6]. Note that the single layer PCMs are not bi-isotropic chiral media and therefore RCP and LCP are not polarisation eigenstates.

#### A simple model for chiral metamaterials

To understand the mechanism of optical rotation and circular dichroism in naturally occurring chiral media, theoretical models were developed on the basis of light-matter interaction using quantum mechanical descriptions [1, 4]. For example, static coupling or one electron theory was developed by Condon, Altar and Eyring [23] and dynamic coupling or coupled oscillator by Oseen [43], Kuhn [44], Boys [45] and Kirkwood [46]. In contrast, for artificial chiral media like metallic helices in epoxy [47], spiral media [48], and PCMs [49], theoretical models have been developed on the basis of resonances in the charge and current induction in metallic elements using the electromagnetic description.

A simple example of the electromagnetic resonance model is the *Ω*-particle model, which describes the resonant induction for a conjugated bilayer gammadion structure [32]. In contrast to typical *Ω*-particles, *Ω*-particles in this model are twisted to take account of chiral nature of geometry as shown in Fig. 3(b). They are also called canonical chiral particles [50] and are thought to be introduced by Jaggard *et al* [51]. Here, the magnetisation **M** is strongly induced by the electric field **E** at ER and the polarisation **P** is strongly induced by the external magnetic field **B** at MR. By solving Maxwell’s equations for the *Ω*-particle, we can obtain the expressions for chirality parameters

(11) $$\begin{array}{@{}rcl@{}} \kappa = \frac{\Omega_{\kappa} \omega_{0} \omega}{{\omega_{0}^{2}} - \omega^{2} - i\omega \gamma}, \end{array} $$

where *Ω*_*κ*_=*μ*_0_ *c*_0_ *α* *A* *N*/*V*_0_ *ω*_0_, $\omega _{0}=1/\sqrt {LC}$, *α*=*l*/*L*, *A* is the cross-sectional area of loop, *V*_0_ is the volume of the unit cell, *N* is the number of resonators in one unit cell, *L* is the inductance and *γ* is the dissipation constant. The details of derivation can be found in the appendix of Ref. [32]. These expressions provide us with important information on how to control the chirality through geometric parameters. We can immediately see that the resonance strength *Ω*_*κ*_ increases as the area *A* or the length *l* increases. This is because the induced magnetic (electric) dipole moments are proportional to *A* (or *l*). In addition, *Ω*_*κ*_ is linearly proportional to *N*/*V*_0_ suggesting that the resonance strength gets enhanced as the density of resonators, i.e. *Ω*-particles, becomes larger.

### Control of optical activity

#### Negative refractive index due to strong optical activity

**Fig. 4:**
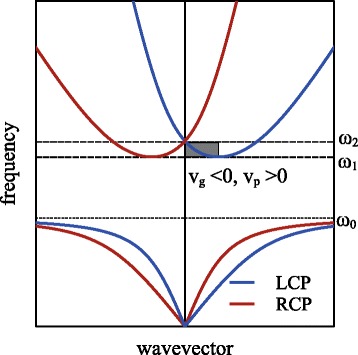
Negative refractive index of chiral metamaterial. Band diagram of chiral metamaterial with a resonator with resonance frequency *ω*_0_ [5]. In the grey region between *ω*_1_ and *ω*_2_, negative refraction can be achieved for the LCP waves since the group velocity (*v*_*g*_) and phase velocity (*v*_*p*_) have opposite signs

Creating a metamaterial that exhibits a negative refractive index [5] has been a major driving force behind research on strong optical activity. Figure 4 shows a dispersion relation of a chiral metamaterial with a resonator. One can see that there is a negative group velocity regime between the frequencies *ω*_1_ and *ω*_2_. Here, the negative refractive index arises from phase velocity and group velocity having opposite directions to each other. The modes with a negative refractive index are often called back propagating waves since the propagation directions of their energy and their phase are opposite. As mentioned in the previous section, optical activity can be dramatically enhanced in chiral metamaterials or nanoplasmonic structures. This implies that the electric field direction of linearly polarised light can be rotated in a very short distance while passing a chiral medium.

**Fig. 5:**
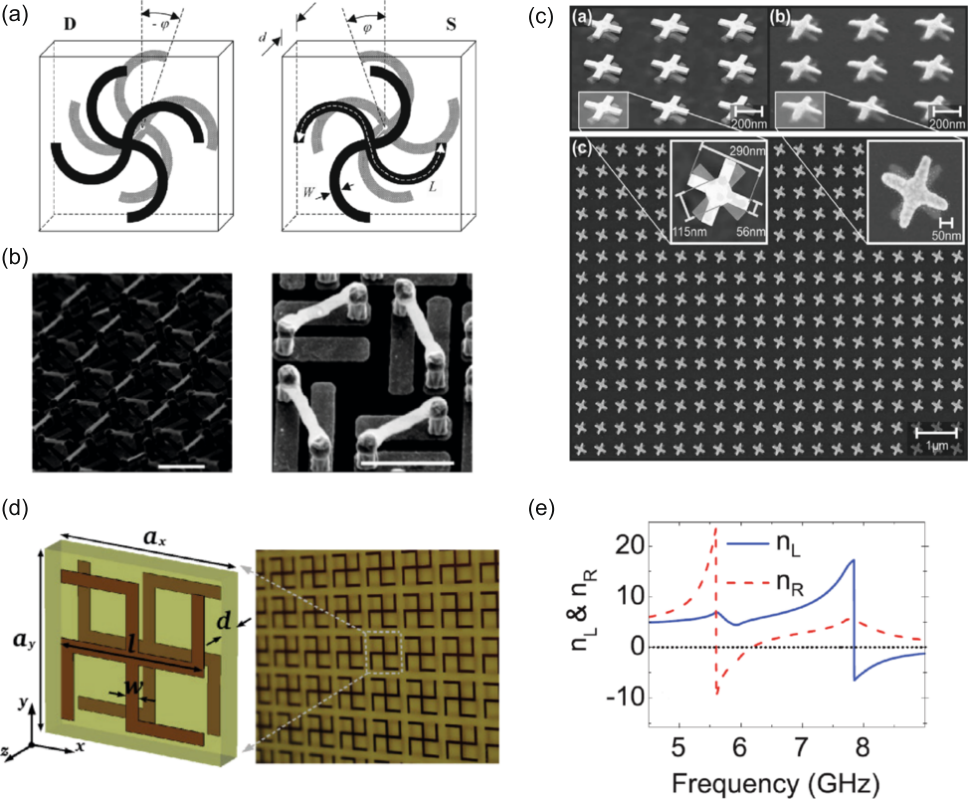
Demonstration of negative refractive index of chiral metamaterial. **a** Dextral (**D**, right-handed) and sinistral (**S**, left-handed) enantiomeric bilayer twisted Rosette structures [3]. **b** Chiral resonator array made of gold. The bottom strips make an angle 29.25° with the top bridge. The size of the unit cell is 40 *μ*m by 40 *μ*m and the scale bars are 20 *μ*m [12]. **c** Schematics (top left) and SEM image (top right, bottom) of an array of right-handed twisted gold crosses [11]. **d** Schematics of a conjugated bilayer gammadion structure [49] **e** Calculated refractive indices for RCP and LCP waves for a conjugated bilayer gammadion structure [49]. The refractive index for RCP *n*_*R*_ becomes negative at around 5.6 GHz

To obtain large optical activity in chiral metamaterials, a variety of designs have been proposed. In 2005, Kuwata-Gonokami *et al.* reported giant optical activity in a single layer gammadion array [6] which results from the enhancement of light-matter interaction through surface plasmons. A large phase shift that led to the giant optical activity was attributed to the slow velocity of surface plasmons compared to that of photons. Rogacheva *et al.* used a bilayer chiral structure (double twisted rosettes) to show a giant polarisation rotation [3] (Fig. 5(a)). In that work, to break mirror symmetry with respect to the middle plane, they twisted the rosettes mutually around the axis of fourfold symmetry. Subsequently, bilayer chiral structures have been widely used to enhance chiral responses. For example, Decker *et al.* demonstrated strong circular dichroism using a double-layered gammadion array on a substrate [52]. There the circular dichroism becomes stronger than that of a single-layer gammadion array owing to a magnetic resonance with currents flowing in opposite directions in each metal layer.

In 2009, Shuang *et al.* used chiral resonator array made of gold to demonstrate negative refractive index [12] as shown in Fig. 5(b). At the same time, an arrangement consisting of a double-layer array of twisted crosses representing an effective form of bilayer chiral structure, has also been studied intensively. For example, Decker *et al.* showed a strong optical activity using a twisted crosses array [11] (Fig. 5(c)) and Dong *et al.* reported a negative refractive index due to a strong chirality parameter of a twisted crosses array [8]. Li *et al.* studied a strong coupling between adjacent layers of twisted crosses [53]. In 2011, Zhao *et al.* proposed a conjugated gammadion array which broke a mirror symmetry along the plane normal direction by placing two gammadion structures that are conjugated to each other on both sides of a dielectric slab [42] (Fig. 5(d)). Since this conjugated gammadion array has inversion symmetry, the chiral response can be more easily understood and can be expressed using the *Ω*-particle model. As shown in Fig. 5(e), a strong chiral metamaterial can have a negative refractive index for one circular polarisation around resonance frequencies. In addition, Li *et al.* used 4-U SRR structure composed of four SRR resonators on a dielectric substrate [54] and comparative study on these designs can be found in the literature [49, 55].

**Fig. 6:**
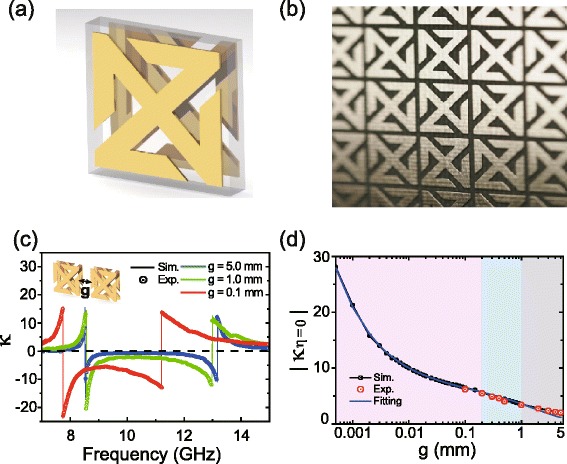
Enhancing optical activity by strong inter-molecular coupling. **a** Unit cell of a conjugated Double-Z metamaterial (CDZM). **b** A photograph of CDZM metamaterial composed of copper and teflon substrate. **c** Retrieved chirality parameters for the gap width *g* of 5.0, 1.0, 9.3 mm. **d** Chirality parameter at zero ellipticity |*κ*_*η*=0_| as a function of the gap width. Adapted from Ref. [10]

Recently, Kim *et al.* used a conjugated double Z metasurface (CDZM) (shown in Fig. 6(a)) and exploited the local field enhancement caused by nanoscopic gaps between metals to enhance their optical activity [10]. In order to enhance the strong inter-molecular capacitive coupling, a conventional conjugated gammadion structure was morphologically transformed into the CDZM. The conjugated arrangement of the CDZM maintains the resonant chiral properties of the conjugated gammadion structure, exhibiting two distinct fundamental resonances in the low frequency range. As shown in Fig. 6(b), the gaps between metallic elements of adjacent unit cells are made very small to enhance the inter-molecular coupling, which can be characterised by an electric capacitance. Then the increased capacitance can draw more electrons [56] thereby increasing the electro-magnetic coupling. Moreover, the CDZM can not only enhance the optical activity but also reduce the ellipticity. Another way of enhancing the optical activity is to increase the number of electric dipoles, for example by increasing the number of arms [57].

#### Bandwidth and dispersion of chiral metamaterials

For practical applications, it is important to control the bandwidth and dispersion of chiral metamaterials. This comes with a challenge because chiral metamaterials normally rely on resonances for a strong optical activity or strong circular dichroism and thus only ‘work’ within a limited frequency range. The dispersion is also strongly affected by the resonant features of chiral metamaterials. Therefore, by necessity, there is a compromise between enhanced optical activity (circular dichroism) and broad bandwidth.

**Fig. 7:**
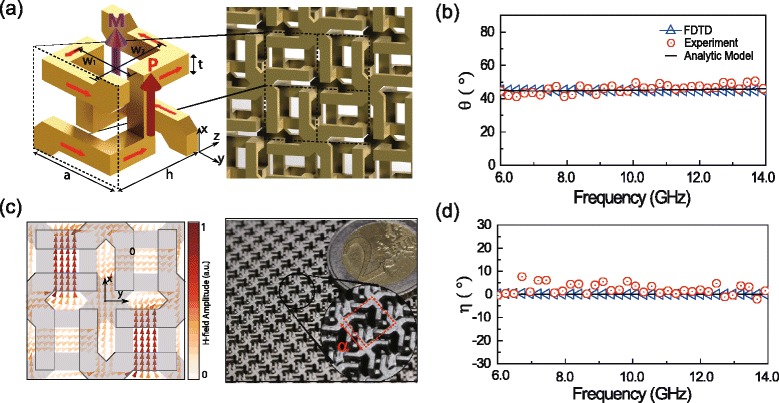
Dispersionless ellipticity. **a** A unit cell of a meshed helical metamaterial. At electrical resonance (ER), the incident electric field (**E**) induces a strong magnetisation (**M**) field in the same direction. **b** Polarisation angle obtained by FDTD simulations, measurement and analytical model. **c** Simulated magnetic field in the unit cell and a photograph of fabricated sample. **d** Ellipticity obtained by FDTD simulations, measurement and analytical model. The meshed helical metamaterial shows nonresonant feature in chirality parameter spectrum with nondispersive ellipticity [59]

Hannam *et al.* [58] demonstrated experimentally large, spectrally flat optical activity and very low ellipticity using a metasurface composed of twisted pairs of metaatoms with their complements. Nondispersive optical activity can also be achieved using meshed helical metamaterials (see Fig. 7(a)) by exploiting a Drude-like response that is nonresonant [59]. As shown in Fig. 7(b)(d), the meshed helical metamaterials shows constant rotation angles and broadband zero ellipticity. As mentioned in Section 2.1.4, the Drude-like response comes from the connectivity over the unit cells. Remarkably, despite the nonresonant features, the meshed helical metamaterial shows strong magnetisation induction as shown in Fig. 7(c) leading to relatively large chirality parameters.

#### Strong circular dichroism

Together with optical activity, circular dichroism has also been of interest in chiral metamaterials. Since material absorption can be significantly increased due to a resonance effect in composite materials, circular dichroism, different absorption of RCP and LCP waves, can be strongly enhanced in chiral metamaterials. However, for practical reasons – measuring circular dichroism is easier for analysing chemical composition, optical activity is useful for photonic applications – circular dichroism has been investigated extensively in nanoplasmonic structures while optical activity has been a particular focus of studies in the microwave and terahertz frequency regimes.

In metal nanoparticle assemblies with a chiral arrangement, optical activity and circular dichroism can also be observed. In a theoretical model developed by Govorov *et al.* [60, 61], analytical expressions for circular dichroism were derived by considering dipolar and multipole Coulomb interactions between nanoparticles. Remarkably, Shen *et al.* [62] have successfully employed chiral DNA structures as a template to fabricate ensembles of randomly oriented three-dimensional plasmonic chiral tetramers with circular dichroism. Moreover, Schreiber *et al.* [63] demonstrated switchable and enhanced circular dichroism by aligning the DNA-origami-scaffold nanoparticle helices.

### Dynamic control of chiral metamaterials

Dynamic control of optical activity and ellipticity is of great interest since it can allow the realisation of new types of switching devices that control polarisation states in real-time. To do this, several different approaches have recently been proposed. In 2009, Kanda *et al.* demonstrated the control of optical activity of a single layer gammadion metamaterials using infrared light [64].Zhou *et al.* showed a larger rotation angle and wider tuning range using a conjugated double layer gammadion structure [65]. More recently, Zhang *et al.* showed a reversal of circular dichroism using a metamolecule consisting of two different metaatoms, one of which has tunable resonance frequencies [17].

### 3D optical chiral metamaterials

#### 3D chiral metamaterials

**Fig. 8:**
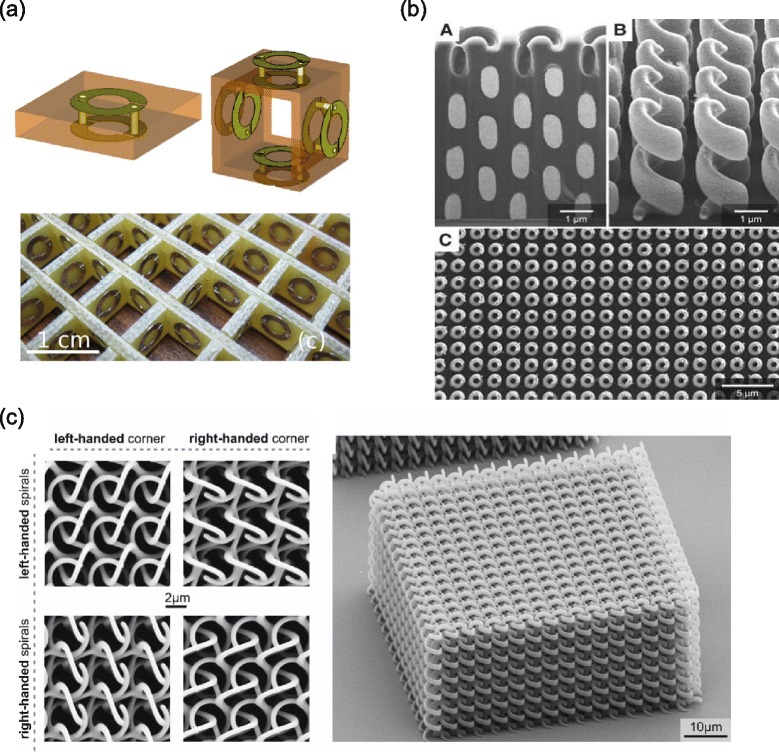
3D chiral metamaterials. **a** Schematic figure (top) and photograph (bottom) of 3D isotropic chiral metamaterials composed of chiral SRR [66]. **b** Two-dimensional array of metallic helices [39]. **c** SEM image of 3D bi-chiral photonic crystals composed of dielectric helices [69]

Fabricating 3D chiral metamaterials is a challenging task, especially for optical wavelength applications, since chiral metamaterials are composed of more complex geometries than achiral metamaterials. Many different structures and fabrication methods have been suggested so far. Among them, the most popular structure is an array of chiral SRRs or helices [66–68]. As shown in Fig. 8(a), Wang *et al.* used a 3D array of chiral split SRRs to fabricate 3D isotropic chiral metamaterials [66]. Gansel *et al.* fabricated a uniaxial photonic metamaterial composed of three-dimensional gold helices (Fig. 8(b)) and studied its optical properties both experimentally [68] and numerically [39]. Their results show that gold helix photonic metamaterials can be used as broadband circular polarisers. Moreover, the circular transmission conversion, i.e. transmission from LCP to RCP or vice versa, is strongly affected by the pitch and radius of helices, wire radius, and the length of helices. Using standard 3D direct laser writing, Thiel *et al.* fabricated bi-chiral dielectric photonic crystals (Fig. 8(c)) composed of three arrays of helices oriented along the three orthogonal directions [69]. This structure shows two distinct types of chirality: one from an arrangement of helices and the other from the chirality of helices. Although this structure is not a 3D metamaterial which has periodicity smaller than the wavelengths, it shows clearly how its geometrical chirality can be associated to optical properties.

Another way of fabricating 3D chiral metamaterials is to use a block-copolymer self-assembly method, a type of bottom-up approach [70, 71]. Using that method, one can fabricate chiral gyroid structures at nanometre scales. In the following section, we will outline some of the interesting features of nanophotonic gyroid structures.

#### Gyroid

**Fig. 9:**
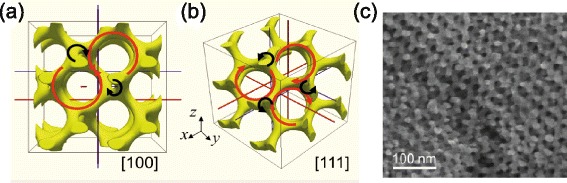
Gyroid metamaterial. **a**, **b** Schematic unit cell of gyroid along the [100] and [110] directions [40]. Along each direction, there are two types of helical structures with different radii and chirality. Larger (smaller) helical structures are denoted by red (black) arrows. **c** SEM image of gold gyroid fabricated using block-copolymer self-assembly method [70]

A single gyroid (SG) metamaterial is a chiral structure that covers constant mean curvature (CMC) domain around a 1*s* *r* *s* network [72, 73]. The shape of the CMC domain is derived from a gyroid surface, a triply-periodic, bi-continuous minimal surface with zero mean curvature. The SG metamaterial is interesting since it can be found in nature in the wing scales of butterfly species [74, 75] and can be fabricated using the block copolymer self-assembly technique [70] which is thought to be a promising candidate for building 3D architectures for nanophotonics [76]. Figure 9 shows a section of 2 ×2×2 unit cells of the SG and the SEM image of the (110) plane of fabricated gold SG. An analytical expression for SG surface reads:

(12) $$\begin{aligned} \sin\left(\frac{2\pi}{a}x\right)\cos\left(\frac{2\pi}{a}y\right)&+\sin\left(\frac{2\pi}{a}y\right)\cos\left(\frac{2\pi}{a}z\right) \\ &+\sin\left(\frac{2 \pi}{a}z\right)\cos \left(\frac{2 \pi}{a}x\right)<t, \end{aligned} $$

where *a* is the periodicity and *t* is the volume fraction parameter (0<*t*<1.5). For example, *t*=1.2 corresponds to a volume fraction of 10 %. As shown in Fig. 9(a)(b), the SG cubic unit cell contains two left-handed screw helices with larger radius (red arrows) and two right-handed screw helices with a smaller radius (black arrows) along the [100] direction, while it has right-handed screw helices with a larger radius (red arrows) and left-handed screw helices with a smaller radius (black arrows) along the [111] direction.

Owing to the development of the block copolymer self-assembly fabrication technique, the gyroid structure has been studied very intensively with both dielectric and metallic forms. A negative refractive index has been theoretically predicted in a metallic gyroid [77], 3D optical metamaterials have been fabricated [70] and the circular dichroism of the metallic gyroid has been analysed using its band structure [40]. On the other hand, it is shown that a dielectric gyroid structure shows a strong circular dichroism due to its band gap [78] and it can be used as a circular polarising beam splitter [79]. For more details on gyroid structures, see the recent review article by Dolan *et al.* [80].

### Nonlinear chiral metamaterials

Freedom in design of metamaterials provides us with an effective way to explore the interesting physical phenomena of “nonlinear chirality”, leading to intensity dependent optical activity [81] and nonlinear magneto-electric responses [82]. In general, nonlinear chirality is very weak. To overcome this, one can use two approaches: (i) using a very strong light source, which is the case in nonlinear optics experiments, and (ii) exploiting local field enhancement, for example surface enhanced Raman spectroscopy and nonlinear plasmonics [83, 84]. To enhance nonlinear chirality for experimental measurements or practical applications, it is essential to control the interaction between the light and nonlinear elements in metamaterials. The light-matter interaction and chirality in metamaterials are very important for optical wavelengths [40, 85]. Recently, Farah *et al.* reported an enhanced ultrafast nonlinear response from a gold gyroid metamaterial, where they showed the plasma frequency shift causes a dramatic enhancement in reflection and absorption upon the injection of a short pulse of pump light [86].

### Photonic topological insulator

One of the most interesting phenomena in condensed-matter physics is the quantum Hall effect in two-dimensional electron systems [87–89]. When a large magnetic field is applied perpendicular to the plane in which the electrons reside, current is carried by electrons along the edges of the system, in so-called chiral edge states. This electronic edge states inspired one-way backward-scattering-immune topological photonic states [90], leading to an experimental verification of the edge states using magnetic photonic crystals [91], coupled ring resonators [92]. A topological insulator is a material with time reversal symmetry and non-trivial topological order in which interfacial electrons can be transported without dissipation even in the presence of imperfections [93]. Interestingly, it behaves as an insulator in the interior but becomes conductive on the surface [94]. Topological photonic states are a novel class of electromagnetic modes that are immune to scattering from imperfections.

**Fig. 10:**
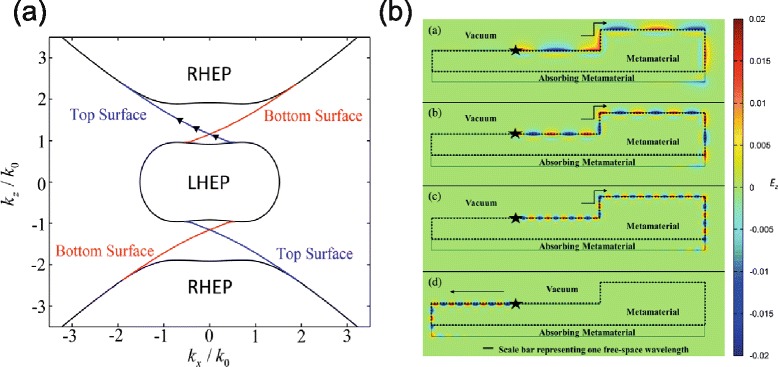
Photonic topological insulator. **a** Topologically protected surface states at the interface between a chiral hyperbolic metamaterial and a vacuum. **b** Cross section view (*x*-*y* plane) of the field distribution. Stars represent line sources with a *z*-dependent phase gradient designed to excite electromagnetic waves with *k*_*z*_=1.1*k*_0_, *k*_*z*_=1.3*k*_0_, and *k*_*z*_=1.5*k*_0_ from the top. The bottom is for a negative chirality parameter *γ*=−0.4 at *k*_*z*_=1.5*k*_0_. Adapted from Ref. [95]

Up until now photonic topological insulators have been implemented on magnetic photonic crystals whose properties are strongly dependent on the periodicity and cannot be described using the effective medium theory. Recently, a new type of photonic topological insulator was proposed combining chirality and hyperbolicity of dispersion relation [95]. In this structure, the backward-scattering-immune one directional state can be observed on its surfaces, which means the light cannot propagate in the interior of medium but can propagate only along its surface. As shown in Fig. 10(a), topologically protected surface states (red and blue lines) exist in the band gap of volume modes (black lines). The modes excited on the surface propagate unidirectionally without experiencing back scattering at corners (Fig. 10).

## Conclusion and Future work

We have reviewed theories and recent efforts to control optical activity and circular dichroism using chiral metamaterials highlighting the recent success in controlling optical properties of the artificial chiral media, both fundamentally and in applications. The enhancement mechanism of optical activity and circular dichroism can be explained theoretically on the basis of electromagnetic resonances. Furthermore, in combination with strong resonances, optical tuning and hyperbolicity, chirality can lead us to a new route to negative refraction, switchable chirality and photonic topological insulators.

Many efforts have been made to fabricate chiral metamaterials using both top-down and bottom-up approaches. Indeed, many recent technologies like direct laser writing and block-copolymer self-assembly methods have brought progress in fabricating 3D visible chiral metamaterials. Nevertheless, it remains challenging to control optical activity and circular dichroism at visible wavelengths due to the limited chiral designs that are experimentally attainable and incomplete control of material properties such as losses and refractive index changes. We believe a combination of smart designs and material property control methods such as (quantum) gain and nonlinearities will provide a new foundation for many practical applications of chirality.
